# Nutritional Intervention of Zinc on Metabolic Disorders Induced by High‐Starch Diets in Largemouth Bass (*Micropterus salmoides*): Insights From Glycogen Metabolism and Insulin Sensitivity

**DOI:** 10.1155/anu/2761195

**Published:** 2026-09-22

**Authors:** Bo Shi, Xiaoxuan Yu, Ziyu Yin, Bo Wang, Jiaming Kang, Baoping Zhang, Zhiyong Dong, Xiaofang Liang, Wanjie Cai, Yuexing Zhang

**Affiliations:** ^1^ National Engineering Research Center for Marine Aquaculture, Marine Science and Technology College, Zhejiang Ocean University, 316022, Zhoushan, Zhejiang, China, zjou.edu.cn; ^2^ Zhejiang Key Laboratory of Deep-Sea Suitable Stock Quality Exploration and High-Efficiency Cultivation Technology, Zhejiang Ocean University, 316022, Zhoushan, Zhejiang, China, zjou.edu.cn; ^3^ Institute of Feed Research, Chinese Academy of Agricultural Sciences, 100081, Beijing, China, caas.cn

**Keywords:** glycophagy, high-starch diet, insulin sensitivity, largemouth bass, zinc

## Abstract

Metabolic disorders induced by high‐starch diets are the primary cause limiting the use of carbohydrates in aquafeeds. This study aimed to investigate the intervention effects of zinc on metabolic disorders induced by high‐starch diets in *Micropterus salmoides*. Five diets were formulated, including a control diet (115 g kg^−1^ starch) and three high‐starch diets (163 g kg^−1^ starch) supplemented with 0 (HS‐LZn), 20 (HS‐MZn) and 150 (HS‐HZn) mg kg^−1^ Zn. After 8 weeks of feeding, intraperitoneal glucose (Glu) tolerance tests (IPGTTs) and intraperitoneal insulin tolerance tests (IPITTs) were conducted to evaluate insulin sensitivity. High‐starch diets inhibited feed intake but improved feed utilization. Diet HS‐HZn significantly increased Zn content in the hepatopancreas. Zn retention decreased significantly as the dietary Zn supplementation level increased from 0 to 150 mg/kg. High‐starch diets significantly improved the retentions of protein and energy. High‐starch diets exerted more pronounced effects on glycogen metabolism and glycolysis. Diets HS‐LZn and HS‐MZn induced severe hepatic glycogen accumulation, concomitant with significant decreases in activities of hexokinase (HK), phosphofructokinase (PFK), pyruvate kinase (PK), glycogen synthase (GS) kinase‐3β (GSK‐3β), and acid alpha‐glucosidase (GAA). Dietary 150 mg/kg Zn supplementation significantly decreased hepatic glycogen and increased activities of HK, PFK, citrate synthase (CS), and GAA. High‐starch diets did not induce severe lipid metabolic disturbances or obvious fat deposition. The insulin tolerance test revealed that the area under the curve (AUC) and incremental AUC (iAUC) for Glu in the HS‐LZn group were significantly higher than the control and HS‐HZn groups. Meanwhile, phosphorylated AKT (p‐AKT) level and immunofluorescence intensity were significantly decreased in the HS‐LZn group, while zinc improved AKT phosphorylation. Untargeted metabolomics analysis demonstrated that high‐starch diets without zinc supplementation inhibited multiple amino acid metabolic pathways.

## 1. Introduction

Carbohydrates are the predominant energy‐yielding nutrients, surpassing fats and protein in energy provision. Indeed, appropriate carbohydrate inclusion in farmed fish diets confers multiple benefits, such as enhancing protein and lipid retentions, decreasing nitrogen loads in aquaculture effluents, and improving feed pellet stability [1]. Nevertheless, given the restricted capacity of fish to metabolize carbohydrate, any further increase in dietary carbohydrate levels must resolve the glucose (Glu)‐induced metabolic disturbances. Among these, glycogenic hepatopathy is especially representative, with its pathological hallmark being excessive glycogen accumulation within hepatocytes [2]. Glycogenic hepatopathy is a potentially reversible condition, for which the diagnostic gold standard is the visualization of intracellular glycogen accumulation in hepatocytes via periodic acid‐Schiff (PAS) staining [3].

Insulin has wide actions in fish, such as feeding, growth, development, and intermediary metabolism [4]. Similar to mammals, it serves as a critical hormone for maintaining Glu metabolic homeostasis. Fish are generally considered Glu intolerant, with high‐starch diets inducing persistent hyperglycemia. Numerous hypotheses exist regarding the physiological limitations of carbohydrate utilization capacity in fish. Among these, insulin resistance is the most predominant and widely accepted hypothesis for Glu intolerance [1]. Insulin resistance is caused by genetic or environmental factors, with dietary composition being the primary contributor. At the cellular level, it manifests as impaired insulin‐mediated Glu uptake and utilization in target tissues, whereas at the molecular level, it is characterized by the inhibition or disruption of insulin signaling pathways [5]. Therefore, alleviating insulin resistance may be an effective approach to improving the carbohydrate utilization capacity in fish.

Zinc is an essential trace element that plays a vital role in metabolism [6]. The most widely recognized function of Zn is its role as a component of metalloenzymes, with numerous enzymes requiring Zn to regulate their activity. Importantly, zinc is involved in insulin secretion and insulin sensitivity. In humans, insulin exists in the form of hexameric crystals, with each hexamer containing two Zn^2+^ and one Ca^2+^ [7]. Zinc is an essential trace mineral required by pancreatic cells for the synthesis, storage, and secretion of insulin [7, 8]. The role of Zn in insulin has been described with various terminologies, including insulin‐mimetic action, insulin‐like activity, and insulin‐sparing effects [7]. Studies in rats have observed that Zn supplementation improved insulin sensitivity [9–14]. Thus, largemouth bass *Micropterus salmoides*, a species highly susceptible to insulin resistance, was selected as the target animal to investigate the intervention effects of zinc on metabolic disorders induced by high‐starch diets.

## 2. Materials and Methods

### 2.1. Experimental Diets

Four iso‐nitrogenous (~530 g/kg) and iso‐energetic (~210 MJ/kg) diets were formulated, including a control diet (11.5% starch) and three high‐starch diets (16.3% starch) supplemented with 0 (HS‐LZn), 20 (HS‐MZn), and 150 (HS‐HZn) mg/kg Zn (ZnSO_4_·7H_2_O as Zn source). The 20 and 150 mg/kg Zn supplementation levels were designed as the optimal level and maximum limit on the basis of “Regulations on the Administration of Feed and Feed Additives” (Regulation [MOARA] No. 2625/2017). This study optimized protein levels by modulating plant‐based protein ingredients (soy protein concentrate, soybean meal, and rapeseed meal) to counteract cellulose‐induced limitations, including shortening the residence time of minerals in the intestine and restricting feed pellet expansion [15–17]. The basal zinc level in the experimental diets was 0.08 g/kg. A similar formulation has also been reported in a previous study on this species [18]. Formulation and nutritional composition of diets are listed in Table S1. For feed processing, refer to our previous study [19].

### 2.2. Fish and Feeding Trial

A total of 600 healthy and size‐graded juveniles (initial weight 83.7 ± 0.05 g) were randomly distributed into 12 tanks (1000 L), with 3 tanks per treatment (*n* = 3) and 50 fish per tank. During the 8‐week trial, fish were hand‐fed three meals per day (8:00, 14:00, and 20:00) to apparent satiation. All uneaten feeds were immediately siphoned out and counted [20]. Water quality parameters, including temperature (25–28°C), dissolved oxygen (> 6.0 mg L^−1^), pH (7.0–7.1), ammonia nitrogen (0.05–0.1 mg L^−1^), and nitrite (0.005–0.025 mg L^−1^) were detected daily.

### 2.3. Sample Collection

Before the feeding trial, 12 fish were kept as 3 initial samples (4 in one). After starving for 24 h, the fish were euthanized with MS‐222. All fish were counted and weighed individually to calculate the growth performance. Ten fish were randomly selected from each tank to measure body length and weights of the whole body, hepatopancreas, gonad, and viscera to determine morphological indexes (CF, HSI, and VSI) and gonad index (GI). Whole‐body compositions were measured from three fish per tank. Hepatopancreas and muscle tissues from four fish per tank were pooled for analysis of Zn, lipid, and glycogen. The serum was obtained from blood samples of 10 fish per tank after centrifugation. Hepatopancreases were collected from six fish per tank for histological and immunofluorescent evaluation. Another piece of hepatopancreas was immediately frozen in liquid nitrogen for mRNA and protein expression analyses and untargeted metabolomics.

### 2.4. Intraperitoneal Glucose Tolerance Tests (IPGTTs) and Intraperitoneal Insulin Tolerance Tests (IPITTs)

The experimental fish recovered for 1 week after the sampling. Fish were fasted for 24 h and then injected intraperitoneally with 1 g Glu/kg and 0.05 mg bovine insulin/kg body weight (Solarbio, China), respectively [21]. Blood was obtained in the basal state (0 min) and 30, 60, 90, and 120 min following Glu or insulin administration. The trapezoidal rule was used to determine the area under the curve (AUC) and the incremental AUC (iAUC).

### 2.5. Chemical Analysis and Biochemical Parameters

Dry matter (AOAC 950.46), crude protein (AOAC 928.08), total lipid (AOAC 991.36), crude ash (AOAC 920.153), and gross energy were determined with the AOAC method [22]. Dietary starch level was determined by the standard methods of GB 5009.9‐2023. Zinc contents in tissues and diets were measured using ICP‐OES (Perkin Elmer, USA). Commercial kits (Nanjing Jiancheng Bioengineering Institute, Nanjing, China) were used to determine the levels of TC, TG, HDL‐C, LDL‐C, total bile acid (TBA), Glu, insulin, and glycogen, as well as activities of hexokinase (HK), pyruvate kinase (PK), phosphofructokinase (PFK), citrate synthase (CS), glycogen synthase (GS), and GS kinase‐3β (GSK‐3β). Glycogen phosphorylase a (GPa) and acid alpha‐glucosidase (GAA) were determined using the ELISA kits (Suzhou Grace Biotechnology Co., Ltd., Suzhou, China).

### 2.6. Histological Examination

Hepatopancreas was fixed by 4% paraformaldehyde, then dehydrated, and embedded in paraffin. Hepatopancreas sections (5‐μm) were stained with oil red O (ORO) and PAS. Images of pathological sections were acquired with an optical microscope (Leica DM2500, Germany). Three random visual fields per sample were examined and quantified with ImageJ software.

### 2.7. Gene Expression

Total RNA was isolated using the Trizol Reagent (Vazyme, China) and then transcribed into cDNA by the kits (Vazyme, China) according to the manufacturer’s protocol. Quantitative real‐time PCR was performed in accordance with standardized methods, employing gene‐specific primers (Table S2). The relative expression levels were normalized by *ef1*α and calculated using the 2^-ΔΔCt^ method, with *E*‐values of 90%–110% for each gene.

### 2.8. Immunofluorescence

Paraffin‐embedded hepatopancreas tissue sections were dewaxed with xylene. After washing, the slices were fixed with 4% paraformaldehyde for 20 min and blocked with 5% BSA for 1 h. Samples were incubated overnight at 4°C with anti‐PI3K (ABclonal, China, 1:200 dilution) and anti‐phosphorylated AKT (p‐AKT, ABclonal, China, 1:200 dilution). Following washing with PBS, the samples were incubated with the secondary antibody (ABclonal, China, 1:500 dilution) for 1 h. DAPI (Beyotime, China) was applied to stain the cell nucleus.

### 2.9. Western Blot

Total protein was extracted from the hepatopancreas using RIPA buffer (EpiZyme, China). Proteins were separated by SDS‐PAGE and subsequently transferred to PVDF membranes. Bolts were blocked with protein‐free rapid blocking buffer (Epizyme, China) for 1 h prior to overnight incubation with primary antibodies (anti‐β‐actin, anti‐AKT, anti‐p‐AKT, and anti‐PI3K, all from ABclonal, China, Table S3) at 4°C, followed by incubation with the IgG‐HRP secondary antibody (ABclonal, China) for 2 h at room temperature. Protein bands were visualized with a luminescent image analyzer (Tanon 5200, China) and quantified with ImageJ software.

### 2.10. Untargeted Metabolomics

The extraction, detection, and quantitative analysis of metabolites were performed by BGI Tech Solutions Co., Ltd. (Shenzhen, China). Approximately 25 mg of the hepatopancreas sample was weighed into a tube with 10 μL of prepared internal standard. After sonication, incubation, and centrifugation, 10 μL of supernatant was filtered for LC‐MS analysis. The Waters UPLC I‐Class Plus (Waters, USA) tandom Q Exactive high‐resolution mass spectrometer (Thermo Fisher Scientific, USA) was used for the separation and detection of metabolites. Chromatographic separation was performed on a Waters ACQUITY UPLC BEH C18 column (Waters, USA). The mass spectrometry data were acquired using the Q Exactive (Thermo Fisher Scientific, USA) and processed with Compound Discoverer 3.3 software (Thermo Fisher Scientific, USA). Metabolite identification was performed by the BMDB (BGI metabolome database), mzCloud, and ChemSpider databases. The results from Compound Discoverer were input into MetaX for data preprocessing, statistical analysis, metabolite classification, and functional annotation. Function annotation was performed through the KEGG database. Metabolites with fold change (FC) ≥ 1.5 or ≤ 0.67 and a *p*‐value ≤ 0.05 were considered differentially abundant metabolites (DEMs). Metabolic pathways with a *p*‐value < 0.05 were defined as significantly enriched pathways.

### 2.11. Statistical Analysis

All data were expressed as the mean and SEM (*n* = 3). Data were checked for a normal distribution and homogeneity of variance before analysis. Statistical analysis was conducted by one‐way ANOVA corrected with Tukey’s multiple comparisons test at a significant level of *p*  < 0.05 (SPSS Statistics 20, IBM, USA). Linear and quadratic effects were assessed using regression analysis.

## 3. Results

### 3.1. Growth Performance and Morphological Indexes

Growth performance and morphological indexes of *M. salmoides* fed diets with different levels of Zn are presented in Table 1. Compared with the control diet, high‐starch diets (HS‐LZn, HS‐MZn, and HS‐HZn) significantly decreased FI and FCR and increased HSI (*p* < 0.05). Meanwhile, HS‐HZn significantly decreased FBW, WGR, and SGR than the control diet (*p* < 0.05). Interestingly, high‐starch diet without Zn supplementation (HS‐LZn) significantly decreased CF compared to the control diet (*p* < 0.05).

**Table 1:** Growth performance and morphological indexes of *M. salmoides* fed diets with different levels of Zn.

Items	Diet				SEM	*p*‐Value		
	Control	HS‐LZn	HS‐MZn	HS‐HZn		*A*	*L*	*Q*
SR (%)	100	100	100	99.3	0.167	0.440	0.192	0.267
IBW (g)	83.8	83.7	83.8	83.7	0.017	0.508	0.586	0.848
FBW (g)	257^a^	252^ab^	253^ab^	249^b^	1.267	0.017	0.031	0.108
FI^1^ (g DM/fish)	176^a^	164^b^	165^b^	161^b^	1.918	0.001	0.002	0.002
WGR^2^ (%)	207^a^	201^ab^	203^ab^	198^b^	1.483	0.040	0.029	0.103
SGR^3^ (%/d)	2.00^a^	1.97^ab^	1.98^ab^	1.95^b^	0.009	0.030	0.035	0.119
FCR^4^	1.02^a^	0.97^b^	0.97^b^	0.97^b^	0.007	0.001	0.008	<0.001
HSI^5^ (%)	1.32^b^	1.63^a^	1.61^a^	1.60^a^	0.040	<0.001	0.008	<0.001
VSI^6^ (%)	7.68	7.92	7.76	7.74	0.044	0.258	0.969	0.361
CF^7^ (g/cm^3^)	2.83^a^	2.65^b^	2.73^ab^	2.74^ab^	0.023	0.021	0.449	0.067
GI^8^ (%)	0.25	0.24	0.27	0.24	0.008	0.049	0.940	0.964

*Note:* Data are presented as mean and S.E.M (*n* = 3), and different superscript letters indicate statistically significant differences (*p* < 0.05, one‐way ANOVA). *A*, one‐way ANOVA; *L*, linear regression analysis; *Q*, quadratic regression analysis.

Abbreviations: CF, condition factor; FBW, final body weight; FCR, feed conversion ratio; FI, feed intake; GI, gonad index; HSI, hepatosomatic index; IBW, initial body weight; SGR, specific growth rate; SR, survival rate; VSI, viscerosomatic index; WGR, weight gain rate.

^1–8^The calculation formulas are listed in the Supporting Information.

### 3.2. Zinc Content, Proximate Composition, and Nutrient Retention

**Figure 1:**
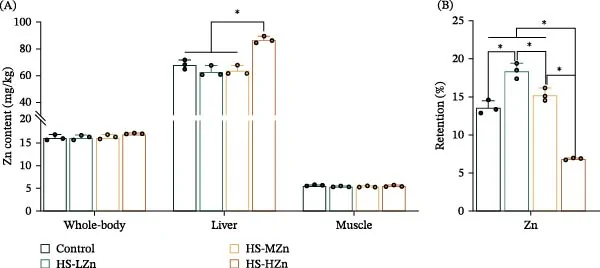
Zn contents in the whole‐body, hepatopancreas, and muscle (A), and Zn retention (B) in *M. salmoides* fed diets with different levels of Zn. The level of significant difference was set at *p*  < 0.05, *n* = 3 per group. The calculation formula of Zn retention was listed in the Supporting Information. The initial whole body zinc content was 19.2 mg/kg. The asterisk symbols indicate significant differences at  ^∗^ *p* < 0.05.

**Figure 2:**
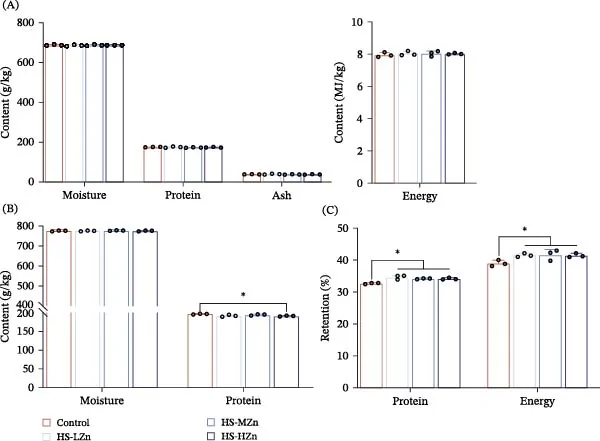
Proximate composition in the whole‐body (A) and muscle (B), and protein and energy retention (C) in *M. salmoides* fed diets with different levels of Zn. The level of significant difference was set at *p*  < 0.05, *n* = 3 per group. The asterisk symbols indicate significant differences at  ^∗^ *p* < 0.05.

Zinc content in tissues and Zn retention are shown in Figure 1. High‐starch diet supplemented with 150 mg/kg Zn (HS‐HZn) significantly increased Zn content in hepatopancreas more than the other diets (*p* < 0.05, Figure 1A). Zn retention decreased significantly as dietary Zn supplementation levels increased from 0 to 150 mg/kg (Figure 1B). As shown in Figure 2, different experimental diets significantly affected protein and energy retention. High‐starch diets significantly increased the retention of protein and energy compared to the control diet (*p* < 0.05, Figure 2C).

### 3.3. Glycogen Metabolism and Glycolysis

**Figure 3:**
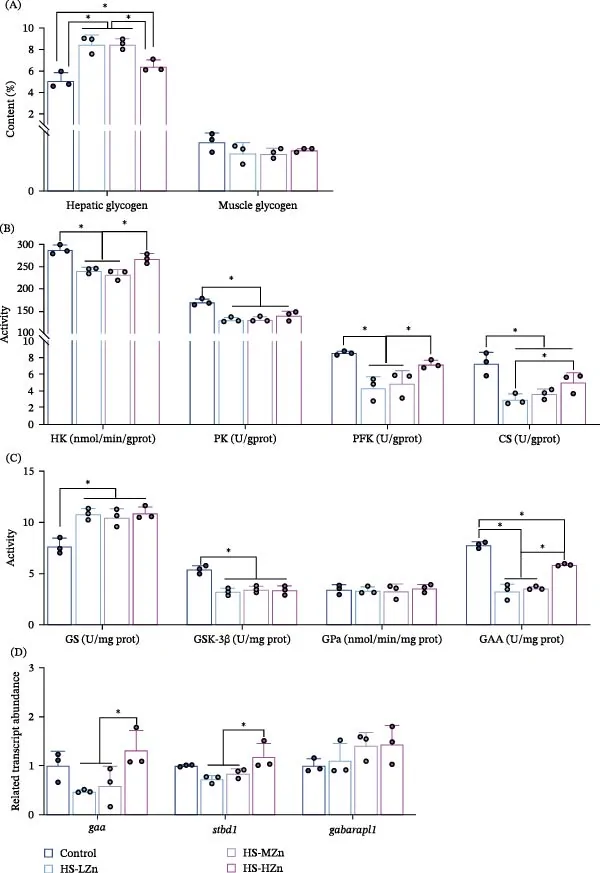
Glycogen metabolism and glycolysis of *M. salmoides* fed diets with different levels of Zn. (A) Glycogen content in the hepatopancreas and muscle. (B) Glycolysis in the hepatopancreas. (C, D) Glycogen metabolism in the hepatopancreas. The significance level was set at *p*  < 0.05, *n* = 3 per group. CS, citrate synthase; GAA, acid alpha‐glucosidase; *gabarapl1*, gamma‐aminobutyric acid receptor‐associated protein; GPa, glycogen phosphorylase a; GS, glycogen synthase; GSK‐3β, glycogen synthase kinase‐3β; HK, hexokinase; PFK, phosphofructokinase 1; PK, pyruvate kinase; *stbd1*, starch binding domain 1. The asterisk symbols indicate significant differences at  ^∗^ *p* < 0.05.

As shown in Figure 3, high‐starch diets supplemented with different Zn significantly affected glycogen metabolism and glycolysis. Hepatic glycogen content in the HS‐LZn and HS‐MZn groups was significantly higher than the control and HS‐HZn groups (*p* < 0.05, Figure 3A). Dietary 150 mg/kg Zn supplementation (HS‐HZn) significantly decreased hepatic glycogen compared with the HS‐LZn and HS‐MZn (*p* < 0.05, Figure 3A). Meanwhile, the activities of HK and PFK in the HS‐LZn and HS‐MZn groups were significantly lower than the control and HS‐HZn groups (*p* < 0.05, Figure 3B). High‐starch diets significantly suppressed PK and CS activities, while HS‐HZn significantly increased CS activity compared with the HS‐LZn (*p* < 0.05, Figure 3B). Compared to the control group, the high‐starch diets significantly enhanced GS activity and inhibited GSK‐3β (*p* < 0.05, Figure 3C). The GAA activity in the HS‐LZn and HS‐MZn groups was significantly lower than the control and HS‐HZn groups (*p* < 0.05, Figure 3C). Fish fed diet with 150 mg/kg Zn (HS‐HZn) significantly upregulated *gaa* and *stbd1* expression compared to the HS‐LZn and HS‐MZn diets (*p* < 0.05, Figure 3D).

### 3.4. Lipid Metabolism

**Figure 4:**
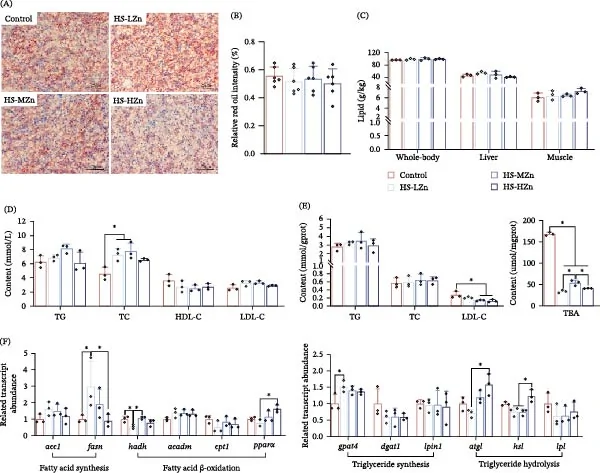
Lipid metabolism of *M. salmoides* fed diets with different levels of Zn. (A, B) Hepatopancreatic histology and original staining area statistics by oil red O staining. (C) Lipid content in tissues. (D, E) Lipid metabolism related parameters in serum and hepatopancreas. (F) Gene expression involved in lipid metabolism. The significance level was set at *p*  < 0.05, *n* = 3 per group. *acadm*, acyl‐CoA dehydrogenase medium chain; *acc1*, acetyl‐CoA carboxylase 1; *atgl*, adipose triglyceride lipase; *cpt1*, carnitine palmitoyltransferase 1; *dgat1*, diacylglycerol oacyltransferase 1; *fasn*, fatty acid synthase; *gpat4*, glycerol‐3‐phosphate acyltransferase 4; *hadh*, hydroxyacyl‐CoA dehydrogenase; HDL‐C, high density lipoprotein cholesterol; *hsl*, hormone‐sensitive lipase; LDL‐C, low density lipoprotein cholesterol; *lpin1*, autosomal recessive lipin‐1; *lpl*, lipoprotein lipase; *pparα*, peroxisome proliferator activated receptor α; TBA, total bile acid; TC, total cholesterol; TG, triglyceride. The asterisk symbols indicate significant differences at  ^∗^ *p* < 0.05.

Histological analysis showed no significant difference in hepatic lipid droplets among fish fed different experimental diets (*p* > 0.05, Figure 4A,B). Meanwhile, no significant differences were observed in lipid contents in the whole‐body, hepatopancreas, and muscle (*p* > 0.05, Figure 4C). Diets HS‐LZn and HS‐MZn significantly increased TC in serum than the control diet (*p* < 0.05, Figure 4D). Fish fed diets HS‐MZn and HS‐HZn showed significantly lower LDL‐C in the hepatopancreas than the control diet (*p* < 0.05, Figure 4E). High‐starch diets significantly decreased TBA in hepatopancreas (*p* < 0.05, Figure 4E). Diet HS‐LZn significantly upregulated mRNA expression levels of *fasn* and *gpat4* and downregulated *hadh* (*p* < 0.05, Figure 4F). The highest expression levels of *pparα*, *atg1*, and *hsl* were observed in HS‐HZn, which were significantly higher than the HS‐LZn (*p* < 0.05, Figure 4F).

### 3.5. IPGTT

**Figure 5:**
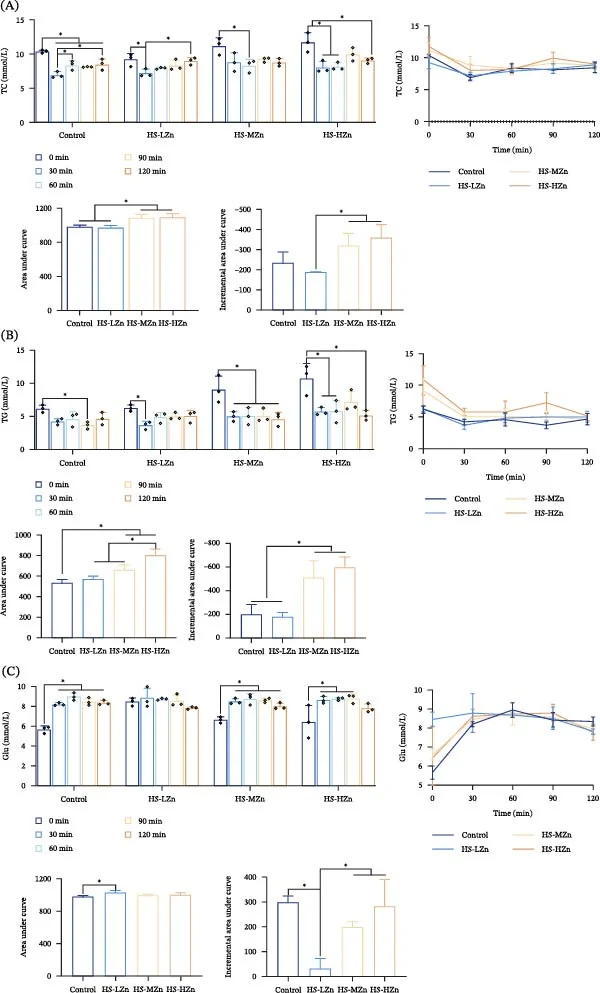
Intraperitoneal glucose tolerance test of *M. salmoides* fed diets with different levels of Zn. (A, B, C) Serum TC, TG, and Glu levels, and the AUC and iAUC. The significance level was set at *p*  < 0.05, *n* = 3 per group. AUC, the area under the curve; Glu, glucose; iAUC, the incremental area under curve; TC, total cholesterol; TG, triglyceride. The asterisk symbols indicate significant differences at  ^∗^ *p* < 0.05.

The results of the IPGTT are shown in Figure 5. Following Glu injection, serum TC and TG levels showed a decreasing trend. Additionally, the AUC and iAUC values for TC and TG were elevated in the HS‐MZn and HS‐HZn groups (*p* < 0.05, Figure 5A,B). The serum Glu level initially increased and then decreased with prolonged injection from 0 to 120 min, peaking at 60 or 90 min postinjection. However, the serum Glu level did not recover to baseline (preinjection) at 120 min (Figure 5C). Furthermore, HS‐LZn significantly increased the AUC for Glu compared with the control diet (*p* < 0.05, Figure 5C). Conversely, the iAUC for Glu in the HS‐LZn group was significantly lower than the other groups (*p* < 0.05, Figure 5C).

### 3.6. IPITT

**Figure 6:**
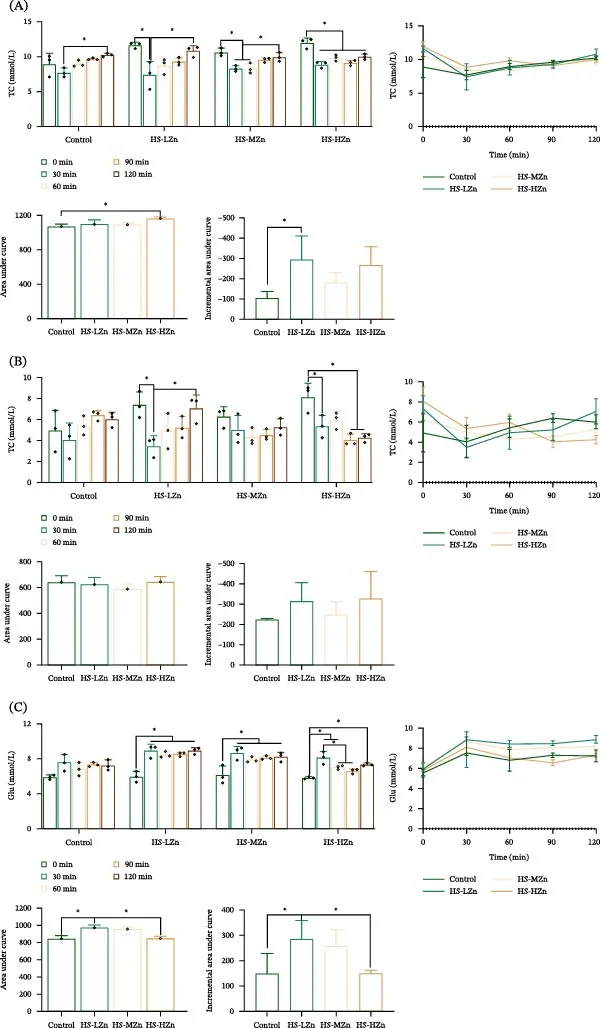
Intraperitoneal insulin tolerance test of *M. salmoides* fed diets with different levels of Zn. (A, B, C) Serum TC, TG, and Glu levels, and the AUC and iAUC. The significance level was set at *p*  < 0.05, *n* = 3 per group. AUC, the area under the curve; Glu, glucose; iAUC, the incremental area under curve; TC, total cholesterol; TG, triglyceride. The asterisk symbols indicate significant differences at  ^∗^ *p* < 0.05.

The results of the IPITT are shown in Figure 6. The serum TC level initially decreased at 30 min, followed by a gradual increase with prolonged injection time (Figure 6A). Diet HS‐HZn exhibited higher AUC for TC than the control diet (*p* < 0.05, Figure 6A). While iAUC for TC in the HS‐LZn group was significantly higher than the control group (*p* < 0.05, Figure 6A). Serum TG level showed an irregular fluctuation pattern, with no significant difference in the AUC and iAUC among different groups (*p* > 0.05, Figure 6B). Compared with preinjection levels, serum Glu levels in the HS‐LZn and HS‐MZn groups were significantly increased at 30, 60, 90, and 120 min postinjection (*p* < 0.05, Figure 6C). Compared with the 30 min, serum Glu levels in the HS‐HZn group significantly decreased at 60, 90, and 120 min following injection (*p* < 0.05, Figure 6C). Diet HS‐LZn significantly increased AUC and iAUC for Glu than the control and HS‐HZn diets (*p* < 0.05, Figure 6C).

### 3.7. Insulin Signaling Pathway

**Figure 7:**
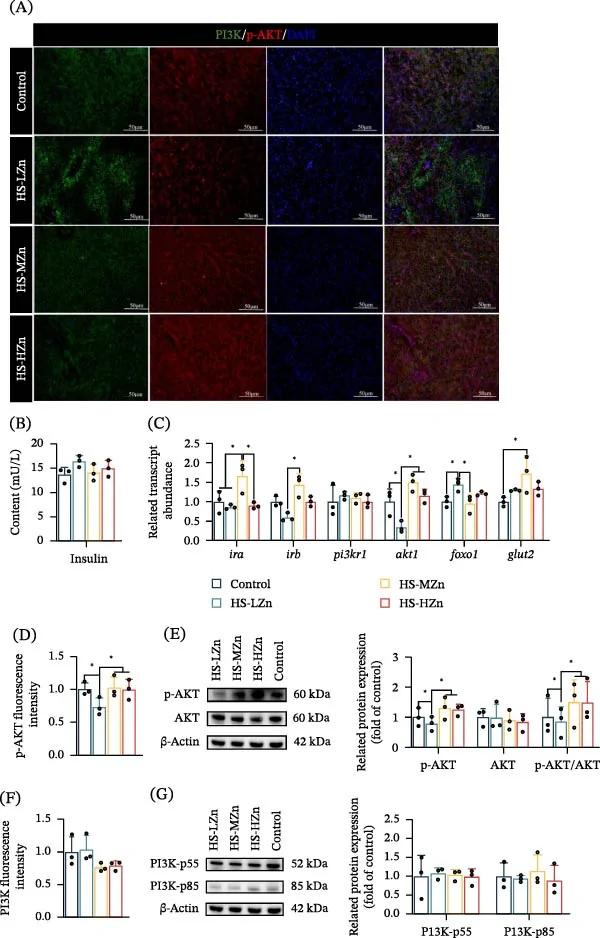
Effects of dietary zinc on insulin signaling pathway. (A, D, F) Representative confocal images and statistics of costaining for PI3K (green) and p‐AKT (red) in hepatopancreas. (B) Level of insulin in serum. (C) Gene expression involved in insulin signaling pathway. (E, G) Hepatopancreatic p‐AKT and PI3K protein expression. The significance level was set at *p*  < 0.05, *n* = 3 per group. *akt1*, serine/threonine kinase 1; *foxo1*, forkhead transcription factor FKHR; *glut2*, SLC2A2 encoding glucose transporter 2; *ira*, insulin receptor a; *irb*, insulin receptor b; *pi3kr1*, phosphatidylinositol‐3‐kinase p85 α. The asterisk symbols indicate significant differences at  ^∗^ *p* < 0.05.

The effects of dietary Zn on the insulin signaling pathway are shown in Figure 7. Costaining of PI3K and p‐AKT showed that HS‐LZn inhibited AKT phosphorylation but had no significant effect on PI3K (Figure 7A, D, and F). Consistent results were also observed in the p‐AKT levels, with fish fed HS‐LZn showing lower p‐AKT levels and p‐AKT/AKT ratios than the other diets (*p* < 0.05, Figure 7E). No significant differences were observed in serum insulin or protein expression levels of PI3K‐p55 and PI3K‐p85 (*p* > 0.05, Figure 7B,G). Diet HS‐LZn significantly downregulated the mRNA expression levels of *irb* and *akt1*, while upregulating *foxo1* (*p* < 0.05, Figure 7C). HS‐MZn significantly upregulated the expression levels of *ira* and *glut2* (*p* < 0.05, Figure 7C).

### 3.8. Untargeted Metabolomics

**Figure 8:**
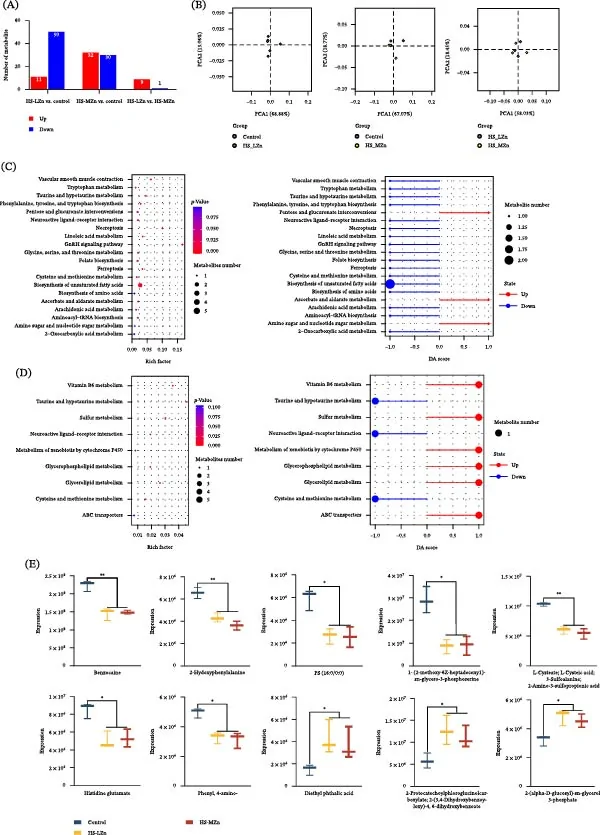
Untargeted metabolomics dataset of *M. salmoides* fed diets with different levels of Zn. (A) The number of significantly different metabolites among three comparison groups (HS‐LZn vs. control, HS‐MZn vs. control, HS‐LZn vs. HS‐MZn). (B) Principal component analysis (PCA) of untargeted metabolite profiles. (C, D) KEGG metabolic pathway enrichment plot (bubble chart) and differential abundance score among two comparison groups (HS‐LZn vs. control, HS‐MZn vs. control). (E) Differentially abundant metabolites shared in two comparison groups (HS‐LZn vs. control, HS‐MZn vs. control). The asterisk symbols indicate significant differences at  ^∗^ *p* < 0.05, and  ^∗∗^ *p* < 0.01.

Pairwise comparison analysis was performed for untargeted metabolomics, revealing 61, 62, and 10 DEMs in the three comparison groups (HS‐LZn vs. control, HS‐MZn *vs*. control, and HS‐LZn vs. HS‐MZn), respectively (Figure 8A). The PCA results showed that three replicate samples from HS‐LZn vs. control and HS‐MZn vs. control clustered distinctly, with a clear separation between different groups. However, the replicate samples from HS‐LZn and HS‐MZn overlapped (Figure 8B). The DEMs in the three comparison groups are shown in Tables S4–S6. Together, twenty and nine significantly enriched pathways were annotated in HS‐LZn vs. control and HS‐MZn vs. control, respectively (Figure 8C,D). The common DEMs between two comparison groups (HS‐LZn vs. control, HS‐MZn vs. control) are shown in Figure 8E, with fish fed the control diet showing higher levels of benzocaine, 2‐hydroxyphenylalanine, PS(16:0/0:0), 1‐(2‐methoxy‐6Z‐heptadecenyl)‐sn‐glycero‐3‐phosphoserine and L‐cysteate; L‐cysteic acid; 3‐sulfoalanine; 2‐amino‐3‐sulfopropionic acid, histidine glutamate, phenyl, 4‐amino‐, and lower diethyl phthalic acid, 2‐protocatechoylphloroglucinolcarboxylate; 2‐(3,4‐dihydroxybenzoyloxy)‐4,6‐dihydroxybenzoate and 2‐(alpha‐D‐glucosyl)‐sn‐glycerol 3‐phosphate than the HS‐LZn and HS‐MZn (*p* < 0.05).

## 4. Discussion

### 4.1. High‐Starch Diets Suppressed Feed Intake, But Improved Feed Utilization and Protein Retention

Starch is a widely used carbohydrate and serves as a staple component in feed formulation for intensive farming operations. However, long‐term consumption of a high‐starch diet imposes a serious burden on the body, leading to metabolic disorders and various diseases [23, 24]. In studies on largemouth bass, it is widely recognized that dietary starch levels below 10% are well tolerated, whereas levels exceeding this threshold impose additional metabolic stress [18]. In this study, high‐starch diets (HS‐LZn, HS‐MZn, and HS‐HZn) inhibited feed intake as reflected by the reduced FI. The reduced feed intake was probably attributable to postprandial hyperglycemia, which promotes satiety via the hypothalamic feeding center, thereby reducing feed consumption. In addition, high‐starch diets significantly decreased the FCR, suggesting better feed utilization. Considering that weight gain remained largely unchanged or only slightly decreased, the lower FCR in the high‐starch diets was mainly attributable to the substantial reduction in feed intake. Although high‐starch diets reduced feed intake, the energy requirements of fish were nevertheless satisfied. This may be attributable to the inherent property of carbohydrates, which are preferentially oxidized for energy conversion. Following digestion and absorption, carbohydrates are converted to Glu and directly channeled into glycolysis for ATP generation, which is energetically more efficient than that of amino acid catabolism. Similar results had also been reported in *M. salmoides* [25, 26], gibel carp *Carassius auratus* var. gibelio [27] and *Pelodiscus sinensis* [20]. *M. salmoides* fed diets containing 0% and 5% corn starch showed higher FI than those fed diets with higher supplementation levels (10%, 15%, and 20%) [26]. Meanwhile, fish fed diet supplemented with 20% corn starch showed the lowest FCR, which was significantly lower than the 5% and 15% corn starch groups [26]. In addition, WGR of HS‐HZn was lower than that of the control group but had no significant differences compared with the HS‐LZn and HS‐MZn groups. Although the zinc supplementation levels in this study were within the recommended range (Regulation [MOARA] No. 2625/2017), excessive zinc still exerted some negative effects on growth in *M. salmoides*. Furthermore, the present study demonstrated that high‐starch diets exhibited the protein‐sparing effect, as evidenced by the higher protein retention, which was consistent with results reported in tilapia *Oreochromis niloticus× O. aureus* [28], Atlantic salmon *Salmo salar*, and rainbow trout *Oncorhynchus mykiss* [29]. The energy derived from starch supports daily maintenance requirements, thereby promoting greater protein deposition in body tissues.

In this study, Zn contents in the whole body and muscle did not show a dose‐dependent increase with dietary zinc levels, implying that these tissues were insensitive to variations in dietary zinc. Conversely, hepatopancreatic Zn increased significantly with graded dietary zinc levels, suggesting that the hepatopancreas was the primary target organ for zinc accumulation. However, Zn retention decreased with increasing dietary Zn levels, which was consistent with our previous findings on zinc [30, 31]. This finding partially reflects the saturable absorption of zinc and a homeostatic regulatory mechanism that prevents potential zinc toxicity.

### 4.2. High‐Starch Diets Induced Severe Glycogen Accumulation, and Zinc Was Associated With Enhanced Glycophagy

High‐starch diets provide sufficient nonprotein energy, exerting a protein‐sparing effect. However, due to the limited capacity of fish to utilize carbohydrates, excess energy cannot be effectively utilized, leading to a series of metabolic disorders [32]. In this study, hepatic glycogen content significantly increased in the HS‐LZn and HS‐MZn groups, accompanied by elevated GS activity and reduced GSK‐3β. Meanwhile, activities of glycolysis rate‐limiting enzymes (HK, PK, and PFK) decreased, suggesting that high‐starch diets induced abnormal accumulation of hepatic glycogen and inhibited glycolysis. This phenomenon has also been reported in a previous study on *M. salmoides* [26]. When fish consume excessive carbohydrates, Glu enters hepatocytes and converts into Glu‐6‐phosphate, which is preferentially channeled into glycogen synthesis, thereby exerting feedback inhibition on glycolysis [26]. Meanwhile, excess energy provided by high‐starch diets causes fish to favor energy storage (glycogen synthesis) over energy utilization (glycolysis), as evidenced by the decreased CS activity in the high‐starch diets. Interestingly, a high‐starch diet supplemented with 150 mg/kg Zn (HS‐HZn) reduced hepatic glycogen. Therefore, we further analyzed the activities of key enzymes involved in the two pathways of glycogenolysis. The phosphorolysis pathway mediated by GPa (it is the active form of GP) is the most classic pathway of glycogen breakdown, primarily involved in rapid energy supply [33]. The glycophagy pathway, with STBD1 serving as the glycogen recognition protein and GAA as the mediating enzyme, is primarily responsible for clearing excess glycogen [34]. The results of this study showed that no significant difference was observed in GPa activity, indicating that the glycogen phosphorolysis pathway remained essentially unchanged. Conversely, different experimental diets significantly affected the GAA activity, indicating alterations in the glycophagy pathway. Diet HS‐HZn significantly increased the activities of GAA, HK, PFK, and CS and upregulated the mRNA expression of *gaa* and *stbd1*, suggesting that zinc potentially activated glycophagy and promoted glycolysis. Further studies are required to uncover the intrinsic mechanisms by which zinc regulates glycophagy and glycolysis.

The present study showed that high‐starch diets did not cause abnormal lipid accumulation in tissues or elevated TG level, a biomarker of abnormal lipid metabolism. Meanwhile, high‐starch diets did not induce significant changes in the expression of genes involved in fatty acid or triglyceride metabolism, with the exception of several alterations attributable to zinc supplementation. Collectively, these findings indicated that high‐starch diets did not cause severe lipid metabolic disorders. This phenomenon has also been observed in the previous research concerning high‐starch diets for *M. salmoides* [35–37]. Following high‐starch intake, fish prioritize the utilization of excess Glu for glycogen synthesis, with the liver demonstrating a robust glycogen storage capacity that can reach several‐fold the normal level. In addition, serum TBA levels did not continuously increase with increasing dietary zinc levels, whereas a significant decrease was observed in the HS‐HZn group. This suggests that the modulatory effect of zinc on bile acid metabolism is not a simple linear response, with high doses potentially exerting an inhibitory effect.

### 4.3. Zinc Enhanced AKT Phosphorylation and Had the Potential to Improve Insulin Sensitivity

This study evaluated the effects of Zn on insulin sensitivity in high‐starch diets through the IPGTT and IPITT, combined with the key proteins in the insulin signaling pathway. The results of IPGTT revealed that the HS‐LZn group exhibited a significantly lower iAUC for Glu than the other groups. Nevertheless, the lower iAUC in HS‐LZn warrants cautious interpretation, given that its baseline Glu level was significantly higher than the other groups. Consequently, the lower iAUC in this group should not be directly interpreted as an enhancement of the Glu clearance ability. In addition, the increased AUC and iAUC values for TC and TG in the HS‐MZn and HS‐HZn groups suggested that Glu loading may have induced lipid fluctuations. Unlike mammals, teleost fish exhibit inherently low insulin sensitivity in peripheral tissues, leading to a markedly lower rate of insulin‐mediated Glu clearance. A previous study has demonstrated that exogenous insulin injection typically caused only a modest reduction in blood Glu levels in rainbow trout [38]. Likewise, no clear Glu‐lowering response was observed following the insulin injection in the present study. Notably, the Glu AUC and iAUC following insulin injection were significantly lower in the HS‐HZn group than in the HS‐LZn and were comparable to those in the control group, indicating that zinc supplementation may exert a beneficial effect on insulin sensitivity.

Zinc has been proven to improve insulin sensitivity by participating in insulin secretion and action [8]. Zinc deficiency impaired insulin secretion and altered insulin‐regulated metabolic signaling [11, 13]. This study showed that the phosphorylation level and immunofluorescence intensity of p‐AKT, a key protein kinase in the insulin signaling pathway, were significantly higher in the HS‐MZn and HS‐HZn groups than the HS‐LZn group, while total AKT levels remained unchanged among treatments. This finding indicated that zinc enhanced AKT phosphorylation, further supporting its positive role in improving insulin sensitivity.

### 4.4. High‐Starch Diets Without Zinc Supplementation Inhibited Multiple Amino Acid Metabolic Pathways

Metabolites are generally regarded as small molecules involved in cellular reactions, providing functional signatures of phenotypes that complement the upstream biochemical information derived from genes, transcripts, and proteins [39]. Metabolomics is the comprehensive study of metabolites and is considered a promising technology for discovering disease biomarkers due to the close relationship between the metabolome and phenotypes [40]. In the present study, 61, 62, and 10 DEMs were detected in the three comparison groups (HS‐LZn vs. control, HS‐MZn vs. control, and HS‐LZn vs. HS‐MZn), revealing that high‐starch diets had more pronounced metabolic effects on *M. salmoides*. These results were consistent with PCA, showing that HS‐LZn and HS‐MZn separated from the control diet, whereas HS‐LZn and HS‐MZn overlapped. We further mapped all DEMs to the KEGG database to identify significantly enriched pathways, and results showed that diet HS‐LZn inhibited the metabolism of multiple amino acids, including tryptophan metabolism; taurine and hypotaurine metabolism; phenylalanine; tyrosine and tryptophan biosynthesis; glycine, serine, and threonine metabolism; cysteine and methionine metabolism; and biosynthesis of amino acids. The alterations in multiple amino acid metabolic pathways induced by high‐starch diets may contribute to decreased insulin sensitivity. Clinical studies have elucidated the associations between compromised insulin action and alterations in intermediary amino acid metabolism [41]. In the present study, the significantly affected metabolites were predominantly enriched in the amino acid metabolic pathways, which may be partially attributed to the starch content, with variations in plant protein composition also contributing substantially.

## 5. Conclusion

In conclusion, high‐starch diets inhibited feed intake and caused severe glycogen accumulation but improved feed utilization and protein retention. Zinc alleviated abnormal hepatic glycogen storage, potentially through enhanced glycophagy. Furthermore, zinc enhanced AKT phosphorylation, suggesting its potential to improve insulin sensitivity. Untargeted metabolomics revealed that high‐starch diets without Zn supplementation inhibited multiple amino acid metabolic pathways. The variation in plant protein composition is a nonnegligible factor affecting the results of the present study.

## Author Contributions

**Bo Shi:** conceptualization, formal analysis, investigation, resources, supervision, writing – original draft. **Xiaoxuan Yu, Ziyu Yin, Bo Wang, Jiaming Kang, and Baoping Zhang:** formal analysis, investigation. **Zhiyong Dong, Xiaofang Liang, and Wanjie Cai:** formal analysis, investigation, writing – review and editing. **Yuexing Zhang**: conceptualization, investigation, resources, supervision, writing – review and editing.

## Funding

This study was supported by the National Natural Science Foundation of China (Grant 32202947).

## Disclosure

All authors have approved the final version of the manuscript.

## Ethics Statement

This study was approved by the Ethics Committee of the Zhejiang Ocean University, complying with all relevant ethical regulations (2025065).

## Conflicts of Interest

The authors declare no conflicts of interest.

## Supporting Information

Additional supporting information can be found online in the Supporting Information section.

## Supporting information


**Supporting Information** Table S1: Formulation and chemical composition of the experimental diets (dry matter). Table S2: Real‐time quantitative PCR primers. Table S3: Antibody information. Table S4: Differentially abundant metabolites in the HS‐LZn vs. control. Table S5: Differentially abundant metabolites in the HS‐MZn vs. control. Table S6: Differentially abundant metabolites in the HS‐LZn vs. HS‐MZn.

## Data Availability

Data are available upon request from the authors.
