# Supplementary material for: Nutritional Intervention of Zinc on Metabolic Disorders Induced by High‐Starch Diets in Largemouth Bass (Micropterus salmoides): Insights From Glycogen Metabolism and Insulin Sensitivity
Source: Aquac Nutr. 2026 Sep 22;2026:2761195. doi: 10.1155/anu/2761195 (PMC13598439; doi:10.1155/anu/2761195)
Supplement: Supplementary file 1 [file ANU-2026-2761195-s001.docx]

**Table S1**

Formulation and chemical composition of the experimental diets (dry matter)

| Ingredients, % | Diet | | | |
| --- | --- | --- | --- | --- |
|  | Control | HS-LZn | HS-MZn | HS-HZn |
| Peruvian fish meal | 37.0 | 37.0 | 37.0 | 37.0 |
| Blood cell meal | 4.00 | 4.00 | 4.00 | 4.00 |
| Wheat gluten | 4.00 | 4.00 | 4.00 | 4.00 |
| Soy protein concentrate | 6.00 | 17.0 | 17.0 | 17.0 |
| Soybean meal | 23.0 | 10.0 | 10.0 | 10.0 |
| Rapeseed meal | 6.00 | 0 | 0 | 0 |
| Tapioca | 3.00 | 3.00 | 3.00 | 3.00 |
| Wheat flour | 7.00 | 15.0 | 15.0 | 15.0 |
| Fish oil | 4.00 | 4.00 | 4.00 | 4.00 |
| Soybean oil | 3.00 | 3.00 | 3.00 | 3.00 |
| Premix ^1^ | 1.30 | 1.30 | 1.30 | 1.30 |
| Ca (H_2_PO4)_2_ | 1.00 | 1.00 | 1.00 | 1.00 |
| Choline chloride | 0.50 | 0.50 | 0.50 | 0.50 |
| Antioxidants | 0.10 | 0.10 | 0.10 | 0.10 |
| Mould inhibitor | 0.10 | 0.10 | 0.10 | 0.10 |
| ZnSO_4_·7H_2_O ^2^, g/kg | 0.09 | 0 | 0.09 | 0.66 |
| Chemical composition, % |  |  |  |  |
| Dry matter | 92.5 | 91.7 | 93.7 | 93.1 |
| Crude protein | 53.4 | 52.8 | 52.6 | 52.6 |
| Starch | 11.5 | 16.3 | 16.3 | 16.3 |
| Total lipid | 11.5 | 10.7 | 10.7 | 11.1 |
| Crude ash | 11.5 | 10.9 | 10.9 | 11.0 |
| Gross energy, MJ/kg | 21.0 | 20.9 | 20.9 | 20.9 |
| Zn, g/kg | 0.11 | 0.08 | 0.10 | 0.25 |

^1^ Mineral (Zn-free, per kg^-1^): FeSO_4_·7H_2_O 503 mg, MnSO_4_·H_2_O 62 mg, CuSO_4_·5H_2_O 79 mg, KIO_3_ 10 mg, NaCI 102 mg, CoCl_2_·6H_2_O 20 mg, MgSO_4_ 1010 mg, Na_2_SeO_3_ 11 mg. Vitamins (per kg^-1^): A 20,000 IU, D_3_ 1,000 IU, E 200 IU, K_3_ 100 mg, B_1_ 34.7 mg, B_2_ 37.3 mg, B_6_ 28.6 mg, B_12_ 0.4 mg, C 200 mg, D-biotin 1.14 mg, pantothenic acid 124 mg, folic acid 20 mg, nicotinamide 206 mg.

^2^ ZnSO_4_·7H_2_O was supply by the Sinopharm Chemical Reagent Co., Ltd, Shanghai, China, in 99.5% purity.

**Table S2**

Real-time quantitative PCR primers

| Genes | Sequence (5’-3’) | Genebank ID/References |
| --- | --- | --- |
| *acadm* | F: TGGCTGAGATGGCAATGAAGG | [1] |
|  | R: TTGGCGATGGAGGCGTAGTA |  |
| *acc1* | F: ATCCCTCTTTGCCACTGTTG | [2] |
|  | R: GAGGTGATGTTGCTCGCATA |  |
| *atgl* | F: CCATGATGCTCCCCTACACT | [2] |
|  | R: GGCAGATACACTTCGGGAAA |  |
| *cpt1* | F: GGGTTGTGCTACCAAGTTCT | XM_038735790.1 |
|  | R: GCCGATTCTCTTCTGTCTTG |  |
| *dgat1* | F: CACGCCTCTTCTTGGAGAAC | [2] |
|  | R: AATGGTACCCACAGCCAGAC |  |
| *fasn* | F: ATCCCTCTTTGCCACTGTTG | [2] |
|  | R: GAGGTGATGTTGCTCGCATA |  |
| *gpat4* | F: GTGTAAAGGCAGCCATAGCC | [2] |
|  | R: GTGCCCACCAGCATTTTACT |  |
| *hadh* | F: AACAGGCTGGACAGATTTGG | [1] |
|  | R: TCAGGAGGGAATCAAACGTC |  |
| *hsl* | F: ATCAGAGCTGGAGCACCCTA | [2] |
|  | R: GCAGAGGAGAGCAGAAAGGA |  |
| *lpin1* | F: TCCTACGTTCCCGAGAGAAA | [2] |
|  | R: TACGAGGGAACCACTTCCTG |  |
| *lpl* | F: TTCCTCGACCCTCTGAAAGA | [1] |
|  | R: GGAGTCAAGTTTGCCAGGAA |  |
| *pparα* | F: CAGAGTTGACGGAGTTCGCC | MK614719.1 |
|  | R: CCAGGGAGTTGAAGCGTGTG |  |
| *gys2* | F: ACAGGGAGGGAAGAGAAA | XM_038725951.1 |
|  | R: CGTTGAGTCATCCAGCAT |  |
| *ugpase* | F: TTTACTCCTCCAGCGTCC | XM_038737122.1 |
|  | R: CTACATCGCAAAGAAAAAGG |  |
| *gaa* | F: TTGTGGAGGCTGGCTATTGG | XM_038730064.1 |
|  | R: TCTCCACGCCTCAGGGATTA |  |
| *stbd1* | F: GCTGTGCGGGTCTCTAATGA | XM_038733208.1 |
|  | R: CAAAGGTTTCCGCCTCTTGC |  |
| *gabarapl1* | F: CACTGGAGGTGAGGAGAGCA | XM_038711194.1 |
|  | R: TGGGGGAAGGGAGTTGTT |  |
| *pygl* | F: TTTTGGCGATGCCTTATG | XM_038728721.1 |
|  | R: TAAAGTCGTTGGGGGCAC |  |
| *akt1* | F: CTTAATTTACCGCCGAAAC | [3] |
|  | R: CCTCCTTGACAATCACCAC |  |
| *foxo1* | F: CTATGAATGGCCGCTTGCTCA | [4] |
|  | R: TCGTCCATATCCGTTGGTGTTG |  |
| *glut2* | F: GAGCCCACGGTACCTTTACA | [1] |
|  | R: ACAGAGGAGCGGATCAAAGA |  |
| *ira* | F: CCCTTGTATCCCTCTCGTTT | [5] |
|  | R: CCAATTTCCTGTTCCTCTCC |  |
| *irb* | F: TGTGTGTCGGAAATAAAACG | [5] |
|  | R: TCTGCGAACAGTCAGGTAGC |  |
| *pi3kr1* | F: AAGACCTTCCTCATCACGAC | [3] |
|  | R: CCTTCCACTACAACACTGCA |  |

**Table S3**

| Antibody | Dilution | Distributor | Phosphorylation site | Catalogue number |
| --- | --- | --- | --- | --- |
| anti-β-actin | 1:10000 (WB) | Abclonal, Wuhan, China | / | AC026 |
| anti-AKT | 1:10000 (WB)  1:400 (IF) | Abclonal, Wuhan, China | / | A18675 |
| anti-phospho-AKT | 1:800 (WB)  1:200 (IF) | Abclonal, Wuhan, China | Ser473 | AP1453 |
| anti-PI3K | 1:5000 (WB)  1:200 (IF) | Abclonal, Wuhan, China | / | A22996 |
| HRP-conjugated Goat anti-Rabbit IgG (H+L) | 1:10000 (WB) | Abclonal, Wuhan, China | / | AS014 |

Antibody information

**Table S4**

Differentially abundant metabolites in the HS-LZn *vs.* Control

| Metabolite description | Fold change | Log_2_(fold change) | *P*-vaule |
| --- | --- | --- | --- |
| ***Up*** |  |  |  |
| Uridine diphosphate glucuronic acid | 6.67 | 2.74 | 0.04 |
| Diethyl phthalic acid | 2.84 | 1.51 | 0.02 |
| 3-Hydroxyisovalerylcarnitine | 2.22 | 1.15 | 0.007 |
| 2-Protocatechoylphloroglucinolcarboxylate; 2-(3,4-Dihydroxybenzoyloxy)-4,6-dihydroxybenzoate; | 2.19 | 1.13 | 0.03 |
| N-lauroyl glycine | 1.64 | 0.71 | 0.03 |
| 4-(trifluoromethyl)benzaldehyde 1-[4-(trifluoromethyl)pyrimidin-2-yl]hydrazone | 1.50 | 0.58 | 0.02 |
| 2-(alpha-D-Glucosyl)-sn-glycerol 3-phosphate; | 1.50 | 0.59 | 0.01 |
| N-(1-benzyl-4-piperidinyl)-4-[(4-chlorophenyl)sulfonyl]-3-methyl-2-thiophenecarboxamide | 1.41 | 0.49 | 0.04 |
| N-Acetyl-alpha-D-glucosamine | 1.25 | 0.32 | 0.04 |
| Pinocembrin 7-O-rhamnoside | 1.24 | 0.31 | 0.03 |
| UVA-1 | 1.24 | 0.31 | 0.03 |
| ***Down*** |  |  |  |
| 1-(4-Methoxyphenyl)piperazine | 0.04 | -4.67 | 0.02 |
| Bioepiderm | 0.25 | -2.00 | 0.02 |
| BB-22 3-Carboxyindole metabolite | 0.25 | -1.99 | 0.01 |
| Norfenefrine | 0.27 | -1.87 | 0.03 |
| 4-Methylumbelliferone | 0.30 | -1.75 | 0.02 |
| 1-(2-methoxy-6Z-heptadecenyl)-sn-glycero-3-phosphoserine | 0.30 | -1.73 | 0.02 |
| Ginsenoside C-K | 0.33 | -1.61 | 0.01 |
| PE(O-16:0/0:0) | 0.35 | -1.50 | 0.03 |
| Peimisine | 0.36 | -1.48 | 0.02 |
| CB-25 | 0.38 | -1.39 | 0.04 |
| 3-(2,7-dimethylpyrazolo[1,5-a]pyrimidin-6-yl)-5-(trifluoromethyl)-4,5-dihydro-1H-pyrazol-5-ol | 0.39 | -1.35 | 0.03 |
| 1H-Indole-2,3-dione | 0.39 | -1.35 | 0.04 |
| N-[1-Hydroxy-3-(morpholin-4-yl)-1-phenylpropan-2-yl]hexadecanamide | 0.39 | -1.35 | 0.04 |
| NP-022229 | 0.40 | -1.32 | 0.05 |
| PC(16:1(9E)/0:0) | 0.45 | -1.17 | 0.05 |
| PS(16:0/0:0) | 0.45 | -1.16 | 0.02 |
| LMST01010328 | 0.45 | -1.15 | 0.03 |
| Cetilistat | 0.47 | -1.09 | 0.03 |
| Ulipristal acetate | 0.48 | -1.06 | 0.02 |
| Cer(d18:0/20:0) | 0.49 | -1.03 | 0.005 |
| Isopilocarpinic acid | 0.49 | -1.03 | 0.03 |
| LysoPC(15:0/0:0) | 0.49 | -1.02 | 0.03 |
| Palmitoleamide | 0.51 | -0.96 | 0.05 |
| Tris(2-butoxyethyl) phosphate | 0.52 | -0.93 | 0.02 |
| PC(16:1(9Z)/P-18:0) | 0.53 | -0.90- | 0.01 |
| PE(0:0/20:1(11Z)) | 0.55 | -0.87 | 0.04 |
| L-Cysteate; L-Cysteic acid; 3-Sulfoalanine; 2-Amino-3-sulfopropionic acid; | 0.58 | -0.78 | 0.009 |
| Methyl 3-(isonicotinoylamino)-4-methylbenzoate | 0.59 | -0.76 | 0.02 |
| Histidine glutamate | 0.59 | -0.75 | 0.02 |
| Spisulosine | 0.61 | -0.71 | 0.01 |
| Estazolam | 0.61 | -0.71 | 0.02 |
| Gly-Pro-Arg-Pro-Lys | 0.62 | -0.70 | 0.01 |
| Benzocaine | 0.65 | -0.62 | 0.01 |
| 2-Hydroxyphenylalanine | 0.66 | -0.60 | 0.004 |
| PC(18:3(6Z,9Z,12Z)/P-18:1(11Z)) | 0.66 | -0.60 | 0.05 |
| Phenyl, 4-amino- | 0.66 | -0.59 | 0.01 |
| 4-Aminobenzoic acid | 0.66 | -0.59 | 0.01 |
| N,N-Dimethyldodecylamine N-oxide | 0.69 | -0.54 | 0.04 |
| Cer(t18:1(6OH)/23:0(2OH)) | 0.71 | -0.49 | 0.02 |
| NP-020480 | 0.75 | -0.41 | 0.02 |
| Docosahexaenoic acid | 0.76 | -0.39 | 0.04 |
| 1-(2-methoxy-octadecanyl)-sn-glycero-3-phosphoserine | 0.76 | -0.39 | 0.04 |
| N'-[(4-Oxo-4H-chromen-3-yl)methylene]nicotinohydrazide | 0.77 | -0.38 | 0.02 |
| 3-methyl-5-oxo-5-(4-toluidino)pentanoic acid | 0.78 | -0.37 | 0.04 |
| Arachidonic acid | 0.80 | -0.32 | 0.03 |
| L-Tryptophan | 0.80 | -0.32 | 0.02 |
| (E,E)-2,4-Decadienoic isobutylamide | 0.81 | -0.30 | 0.05 |
| Carylophyllene Oxide | 0.81 | -0.30 | 0.05 |
| Indoleacrylic acid | 0.82 | -0.28 | 0.01 |
| PC(18:0/18:4(6Z,9Z,12Z,15Z)) | 0.83 | -0.27 | 0.01 |

**Table S5**

Differentially abundant metabolites in the HS-MZn *vs.* Control

| Metabolite description | Fold change | Log_2_(fold change) | *P*-vaule |
| --- | --- | --- | --- |
| ***Up*** |  |  |  |
| Tetradecenoylcarnitine | 2.55 | 1.35 | 0.02 |
| 5-Methyltetrahydrofolic acid | 2.46 | 1.30 | 0.04 |
| Diethyl phthalic acid | 2.45 | 1.29 | 0.04 |
| beta-Cortol | 2.33 | 1.22 | 0.001 |
| Lauroylcarnitine | 2.31 | 1.21 | 0.008 |
| 9-Ethylguanine | 2.28 | 1.19 | 0.04 |
| D-alpha-Hydroxyglutaric acid | 2.02 | 1.01 | 0.04 |
| 4-Pyridoxic acid | 2.01 | 1.01 | 0.008 |
| 2,2'-Azobis(4-methoxy-2,4-dimethylvaleronitrile) | 2.00 | 1.00 | 0.04 |
| 2-Protocatechoylphloroglucinolcarboxylate; 2-(3,4-Dihydroxybenzoyloxy)-4,6-dihydroxybenzoate; | 1.91 | 0.94 | 0.04 |
| 11Z,13E-Hexadecadienal | 1.83 | 0.87 | 0.05 |
| 7-Methoxy-2,3-dihydropyrrolo[2,1-b]quinazolin-9(1H)-one | 1.80 | 0.85 | 0.05 |
| 2-[[9-(1-Methylethyl)-6-[(phenylmethyl)amino]-9H-purin-2-yl]amino]-1-butanol | 1.79 | 0.84 | 0.05 |
| 5-Chloro-4-N-(cyclopropylmethyl)-2-methyl-4-N-propyl-6-N-(2,4,6-trichlorophenyl)pyrimidine-4,6-diamine | 1.79 | 0.84 | 0.003 |
| Glycinamide, glycyl-L-prolyl- | 1.75 | 0.81 | 0.02 |
| 4-(methylthio)-6-phenyl-2-(3-pyridyl)pyrimidine-5-carbonitrile | 1.73 | 0.79 | 0.006 |
| 2-Carboxy-2-hydroxy-8-carboxychromene; | 1.73 | 0.79 | 0.01 |
| DL-Homocysteic acid | 1.72 | 0.78 | 0.03 |
| Nitrosoheptamethyleneimine | 1.56 | 0.64 | 0.03 |
| O-ACETYLCARNITINE | 1.55 | 0.63 | 0.04 |
| Glycerol 3-phosphate | 1.54 | 0.62 | 0.02 |
| NP-021733 | 1.49 | 0.58 | 0.02 |
| trans-Hexadec-2-enoyl carnitine | 1.49 | 0.57 | 0.05 |
| 7-Hydroxymethyl-12-methylbenz[a]anthracene sulfate; | 1.47 | 0.55 | 0.02 |
| Austroyunone A | 1.46 | 0.54 | 0.005 |
| NCGC00380363-01!8-(2,3-dihydroxy-3-methylbutyl)-5,7-dimethoxy-2-phenyl-2,3-dihydrochromen-4-one [IIN-based on: CCMSLIB00000848735] | 1.44 | 0.53 | 0.04 |
| Glycine, L-g-glutamyl-L-cysteinyl-, 3-methyl ester | 1.43 | 0.51 | 0.01 |
| 2-(alpha-D-Glucosyl)-sn-glycerol 3-phosphate; | 1.41 | 0.50 | 0.02 |
| N-(tert-Butyl)-N'-(2-hydroxyethyl)urea | 1.28 | 0.35 | 0.05 |
| Levetiracetam | 1.27 | 0.34 | 0.04 |
| S-(1,2,2-trichlorovinyl)-L-cysteine | 1.26 | 0.34 | 0.01 |
| Polysulfide; (Sulfide)n; Polysulfane; | 1.20 | 0.26 | 0.03 |
| ***Down*** |  |  |  |
| N6-Cyclopentyladenosine | 0.17 | -2.59 | 0.001 |
| (24RS)-28,28,28-trifluoro-25-hydroxyvitamin D2 | 0.20 | -2.31 | 0.04 |
| PA(20:1(11Z)/0:0) | 0.22 | -2.18 | 0.05 |
| LysoPC(14:0/0:0) | 0.22 | -2.16 | 0.03 |
| PC(17:2(9Z,12Z)/0:0) | 0.30 | -1.74 | 0.03 |
| 1-(2-methoxy-6Z-heptadecenyl)-sn-glycero-3-phosphoserine | 0.31 | -1.67 | 0.04 |
| 2-[2,6-dihydroxy-4-({[(3R,4R)-3-(4-hydroxybenzamido)azepan-4-yl]oxy}carbonyl)benzoyl]-3-hydroxybenzoic acid | 0.38 | -1.40 | 0.03 |
| LysoPE(P-16:0/0:0) | 0.38 | -1.40 | 0.02 |
| Bevirimat | 0.41 | -1.30 | 0.002 |
| (2R)-2-{(1R)-2-Oxo-1-[(2-thienylacetyl)amino]ethyl}-5,6-dihydro-2H-1,3-thiazine-4-carboxylic acid | 0.42 | -1.25 | 0.04 |
| PS(16:0/0:0) | 0.43 | -1.22 | 0.04 |
| CMP-2-keto-3-deoxy-D-glycero-D-galacto-nononate; CMP-3-deoxy-D-glycero-beta-D-galacto-non-2-ulosonate; CMP-KDN; | 0.44 | -1.17 | 0.003 |
| ARN-509 | 0.44 | -1.17 | 0.03 |
| SM-17466; | 0.46 | -1.13 | 0.02 |
| Cholylhistidine | 0.49 | -1.03 | 0.03 |
| 5-(1,2,4a,5-tetramethyl-7-oxo-1,2,3,4,4a,7,8,8a-octahydronaphthalen-1-yl)-3-methylpentanoic acid | 0.51 | -0.98 | 0.04 |
| Glycerophospho-N-Arachidonoyl Ethanolamine | 0.52 | -0.93 | 0.05 |
| L-Cysteate; L-Cysteic acid; 3-Sulfoalanine; 2-Amino-3-sulfopropionic acid; | 0.52 | -0.93 | 0.02 |
| Alniditan | 0.54 | -0.89 | 0.02 |
| 2-Hydroxyphenylalanine | 0.55 | -0.86 | 0.003 |
| DG(18:4(6Z,9Z,12Z,15Z)/17:0/0:0)[iso2] | 0.58 | -0.79 | 0.02 |
| [(2R,5R)-5-(2-Amino-6-oxo-1H-purin-9-yl)-3,4-dihydroxyoxolan-2-yl]methyl dihydrogen phosphate | 0.59 | -0.77 | 0.01 |
| PC(O-14:0/16:0) | 0.62 | -0.69 | 0.02 |
| Histidine glutamate | 0.62 | -0.68 | 0.03 |
| Phenyl, 4-amino- | 0.63 | -0.66 | 0.03 |
| Ethionamide | 0.65 | -0.62 | 0.04 |
| 20-HETE ethanolamide | 0.66 | -0.60 | 0.01 |
| Benzocaine | 0.67 | -0.58 | 0.003 |
| PC(22:5(7Z,10Z,13Z,16Z,19Z)/14:0) | 0.81 | -0.31 | 0.01 |
| PC(18:1(9Z)/18:1(9Z)) | 0.81 | -0.31 | 0.03 |

**Table S6**

Differentially abundant metabolites in the HS-LZn *vs.* HS-MZn

| Metabolite description | Fold change | Log_2_(fold change) | *P*-vaule |
| --- | --- | --- | --- |
| ***Up*** |  |  |  |
| C20822; Carbapenem biosynthesis intermediate 3; | 10.43 | 3.38 | 0.03 |
| 4'-Thiothymidine | 4.67 | 2.22 | 0.02 |
| 5-Hydroxy-1-(4-hydroxy-3-methoxyphenyl)-3-tetradecanone | 2.52 | 1.33 | 0.02 |
| Avocadyne 1-acetate | 1.72 | 0.79 | 0.03 |
| PC(O-14:0/16:0) | 1.56 | 0.64 | 0.02 |
| 3-Hydroxyisovalerylcarnitine | 1.50 | 0.58 | 0.03 |
| N-lauroyl glycine | 1.49 | 0.57 | 0.03 |
| 7-O-Demethyl Rapamycin | 1.31 | 0.39 | 0.03 |
| (4S,5S,8S,10R)-4,5,8-trihydroxy-10-methyl-3,4,5,8,9,10-hexahydro-2H-oxecin-2-one | 1.27 | 0.34 | 0.03 |
| ***Down*** |  |  |  |
| N-Methacryloyl-L-glutamic acid | 0.17 | -2.59 | 0.05 |

Calculation formula:

FI = total feed intake per tank/number of fish in tank.

WGR = 100 × (final total weight + total weight of dead fish - initial total weight)/initial total weight.

SGR = 100 × [ln (final mean body weight/initial mean body weight)]/days.

FCR = FI/(final mean body weight - initial mean body weight).

HSI = 100 × (hepatopancreas weight)/(body weight).

VSI = 100 × (viscera weight)/(body weight).

CF = 100 × (body weight)/(body length)^3^.

GI = 100 × (gonad weight)/(body weight).

Retention of nutrient (protein, energy) = 100 × (final body weight × nutrient in the final whole body − initial body weight × nutrient in the initial whole bod)/ nutrient in diet.

Zn retention = 100 × (final whole body Zn content − initial whole body Zn content)/ Zn intake.

References:

1. P. Chen, Y. Zhu, X. Wu, et al., “Metabolic adaptation to high-starch diet in largemouth bass (*Micropterus salmoides*) was associated with the restoration of metabolic functions via inflammation, bile acid synthesis and energy metabolism,” *British Journal of Nutrition* 129 no. 3 (2023): 381-394.

2. H. H. Yu, X. F. Liang, P. Chen, et al., “Dietary supplementation of Grobiotic®-A increases short-term inflammatory responses and improves long-term growth performance and liver health in largemouth bass (*Micropterus salmoides*),” *Aquaculture* 500 (2019): 327-337.

3. S. Li, C. Sang, A. Wang, et al., “Effects of dietary carbohydrate sources on growth performance, glycogen accumulation, insulin signaling pathway and hepatic glucose metabolism in largemouth bass, *Micropterus salmoides*,” *Aquaculture* 513 (2019): 734391.

4. P. Chen, “Mechanism of FoxO1 on Glucose and Lipid Metabolism in *Micropterus salmoides*” Chinese Academy of Agricultural Sciences (2021)

5. S. Li, A. Wang, Z. Wei, et al., “Insulin receptors in largemouth bass (*Micropterus salmoides*): Cloning, characterization, tissue expression profile and transcriptional response to glucose tolerance test,” *Aquaculture Research* 52 no. 2 (2021): 625-634.
